# Surgical histopathologic findings of 232 Chinese children cases with drug‐resistant seizures

**DOI:** 10.1002/brb3.1565

**Published:** 2020-02-14

**Authors:** Yao Kun, Duan Zejun, Zhou Jian, Zhai Feng, Liu Changqing, Qi Xueling

**Affiliations:** ^1^ Department of Pathology SanBo Brain Hospital Capital Medical University Beijng China; ^2^ Department of Neurosurgery SanBo Brain Hospital Capital Medical University Beijng China; ^3^ Department of Functional Neurosurgery SanBo Brain Hospital Capital Medical University Beijng China

**Keywords:** drug‐resistant seizures, focal cortical dysplasia, histopathologic findings, seizure outcome

## Abstract

**Objectives:**

The drug‐resistant seizures are characterized by frequent and severe onset of seizures in childhood. There is only little literature had extensively explored the types of pathological brain damage in Chinese children cases. The present study aims to investigate the histopathologic findings and seizure outcomes of drug‐resistant seizures in cases of 0–14‐year‐old children.

**Materials and Methods:**

About 232 children cases were retrospectively who underwent epilepsy surgery. The medical records, onset age, age of surgery, disease course, seizure type, pathological reports, and seizure outcomes of these patients were retrospectively analyzed.

**Results:**

The most common categories were malformations of cortical development (focal cortical dysplasia (FCD) was the most common type (94.36%, 67/71), found in 30.60% of the patients, tumors in 18.11%, glial scar in 12.50%, and encephalitis in 11.63%). It was found that the effective seizure outcome of FCD cases with shorter duration of epilepsy (<2 years, 87.23%) was better than that with longer duration (≥2 years, 60.00%) and the difference was statistically significant (*χ* ^2^ = 4.76, *p* < .05). Patients with FCD I, FCD II, and tumor showed the relatively better postsurgical seizure outcome than patients with other pathological types.

**Conclusion:**

The malformations of cortical development (MCD) (most FCD) were the most common pathological type for children cases in China with drug‐resistant seizures. It was speculated further that the FCD patients with shorter duration of epilepsy before surgery seem to have a higher ratio of being seizure‐free after surgery.

## Graphical abstract

The malformations of cortical development (most FCD) were the most common pathological type for children cases in China with drug‐resistant seizures. It was speculated further that the FCD patients with shorter duration of epilepsy before surgery seem to have a higher ratio of being seizure‐free after surgery.

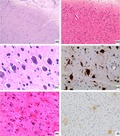

## 1 INTRODUCTION

The drug‐resistant seizures are defined as failure of adequate trials of two tolerated and appropriately chosen and used antiseizure drugs schedules (whether as monotherapies or in combination) to achieve sustained “seizure freedom” (Kwan et al., 2010). It is hard to control by medication and often results in growth and mental retardation in children (Glauser et al., (2006); Larsson & Eeg‐Olofsson, 2006). Surgery was considered as a better resort for the disease because of severe onset and medical treatment failures. Although the efficacy of epilepsy surgery has been established in retrospective series from many literatures (Blumcke et al., 2017; Fauser et al., 2006; Kral et al., 2007; Krsek et al., 2009; Rickert, 2006), few of these studies have extensively explored the pathological subtypes of brain lesions in Chinese children cases. Blumcke et al. (2017) reported the diagnoses made on the basis of resected brain specimens from 9,523 patients (including 2,623 children) who underwent epilepsy surgery for drug‐resistant seizures from 12 European countries. They found in children patients FCD was the most common diagnosis and tumors were the second most common lesion. However, the types of underlying pathologic brain lesions in Chinese children are still lacking. The present study retrospectively analyzed the types of underlying pathologic brain lesions in 232 Chinese children who underwent epilepsy surgery. Besides, seizure outcome was also retrospectively performed which provided better evidence.

## 2 MATERIALS AND METHODS

### 2.1 Patients and samples

All of epilepsy cases who underwent epilepsy surgery were obtained from the pediatric department of functional neurosurgery of San Bo Brain Hospital between June 2008 and December 2012. The medical records, onset age, age of surgery, disease course, seizure type, pathological reports, and seizure outcomes of these patients were retrospectively summarized and analyzed. And all patients' parents had given their informed consent to the study. This research was approved by the ethics committee of San Bo Brain Hospital, Capital Medical University (IRB code 201507).

### 2.2 Preoperative evaluation

All of cases were submitted to go through presurgical assessments using a standard protocol comprising of clinical, neuroradiological, and electroencephalogram (EEG) examination. For each patient, the presurgical evaluation included continuous scalp video‐EEG monitoring, high‐resolution magnetic resonance imaging (MRI), magnetoencephalography (MEG), and neuropsychological assessments. The children in this study had at least one follow‐up at 12 months after surgery, including neurological examination, EEG and MRI assessment, and, if necessary, invasive video‐EEG recordings, neurological and ophthalmological investigations, neuropsychological testing, as well as modern neuroimaging (MRI, PET, SPECT) (Rickert, 2006). For patients with involvement of the functional cortex, functional MRI for evaluation of the motor unit reorganization and the research of the risk of postoperative deterioration were done. All operations are performed by the same neurosurgeon.

### 2.3 Pathological examination and pathological diagnosis

Gross morphology of the fresh brain tissues excised from surgeries was photographed, and tissues were cut perpendicularly to the surface of cortex in a coronal plane at interval of 5 mm. The sliced samples were positioned at the same horizon and direction, and the anatomical plane of tissues was observed and photographed. Samples were fixed in 10% neutral buffered formalin overnight for histological examinations. Furthermore, samples were specially dehydrated, paraffin embedded, and sectioned at 5 µm thickness following the procedures. After that sections were stained with hematoxylin and eosin and/or immunohistochemical stains. Pathological results were analyzed by two experienced specialists in the field of neuropathology.

### 2.4 Criteria for evaluation of therapeutic effects

Postoperative seizure outcome was classified according to Engel: (I) seizure‐free or auras only or convulsions with drug withdrawal only, (II) rare seizures (<2 seizures/year or >90% seizure reduction), (III) reduction in seizure frequency >75%, and (IV) reduction in seizure frequency <75%. Engel I–II class were artificially regarded as effective prognosis and Engel III‐IV as ineffective by us. The postsurgical follow‐up period was at least 12 months. The prognostic rate for seizures was defined as the percentage of cases with effective prognosis (Engel I–II class).

### 2.5 Statistical analysis

Data were analyzed by SPSS Statistics 19.0 software. Statistical significance was examined by use of *χ* ^2^ and Fisher's exact test. *p* < .05 was accepted as statistically significant.

## 3 RESULTS

All of the patients showed drug‐resistant seizures including 143 male cases and 89 female cases. In the 232 cases, the mean age was 4.1 years old (ranged from 0.1 to 14.0). The mean age at surgery was 8.3 years (ranged from 0.1 to 14.0). Focal impaired awareness seizures were the most common seizure type in all of the patients. In most cases, they can develop to generalized tonic–clonic seizures. The pathology of the specimens resected was summarized (Table 1).

**Table 1:** Proportions for each pathological type of refractory epilepsy in all of the cases

Pathological type	Number of cases	Proportion (%)
FCD	67	28.86
FCD I a	12	5.17
FCD I b	14	6.03
FCD II a	14	6.03
FCD II b	27	11.63
Gyrus malformation	4	1.73
Hippocampal sclerosis	13	5.61
Tumor	42	18.11
Vascular malformations	0	0
Glial scar	29	12.50
Encephalitis	27	11.63
Tuberous sclerosis	9	3.87
No obvious pathological abnormality	23	9.92
Hydatoncus	1	0.44
Heterotopias	3	1.29
Hamartoma	7	3.02
Sturge–Weber syndrome	7	3.02
Total	232	100%

### 3.1 Malformations of cortical development (MCD)

**Figure 1:**
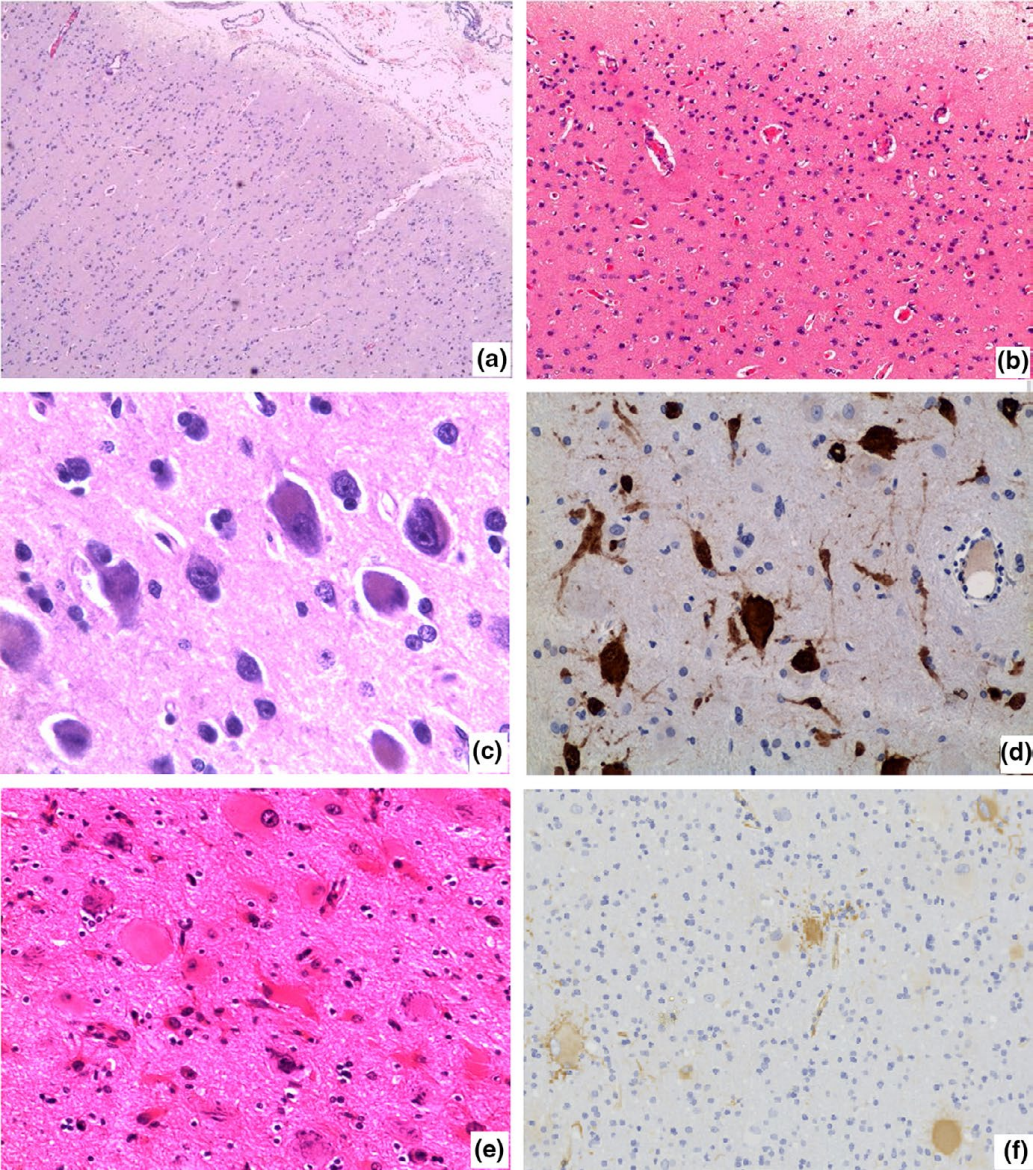
Histopathology of FCD type I and FCD type II. (a) Abnormal cortical architecture with columnar disorganization (HE, ×100); (b) abnormal cortical architecture with dyslamination disorganization (HE, ×200); (c) neurons with abnormal morphology and nucleus (HE, ×400); (d) neurons with abnormal morphology marked by NF (×400); (e) BCs: the nucleus were offset or absent, and excess cytoplasm was stained in homogeneous pink (HE × 400); (f) Nestin staining of BCs (×400)

Malformations of cortical development were found in 30.59% (71/232) of specimens and were the most common histopathologic category; subtypes of FCD were the most common MCD, accounting for 94.36% (67/71) of cases. Focal cortical dysplasia malformations were characterized by architectural and cytoarchitectural abnormalities of the six layers of the cerebral cortex. The FCD I series, accounting for 38.80% (26/67) of FCD cases (Table 1), with pathology as previously described (Fauser et al., 2006), type I focal cortical dysplasias into type Ia, marked by isolated architectural abnormalities (Figure 1a,b), and type Ib, characterized by architectural abnormalities plus giant or immature neurons. In the 26 FCD I cases, patient detailed clinical characteristics were seen in Table 2. Thirteen patients had lesions in the temporal lobe, accounting for 50% of all cases. Postsurgical follow‐ups found that 19 cases had good seizure outcomes (Engel I:13, Engel II:6) and 7 cases had poor seizure outcomes. The effective prognostic rate was 73.07% (Table 2).

**Table 2:** Major histopathologic disease categories among children

Pathological type	Number of cases	Sex		Mean age at epilepsy onset/range	Mean age at surgery/range	Mean duration of epilepsy before surgery/range	Outcome (prognostic rate) (%)
		M	F				
FCD I	26	15	11	2.3/0−3–10.8	7.1/2.3–14.0	4.8/0.2–13.5	73.07
FCD II	41	27	14	2.0/0.1–11.9	6.1/1.6–14.0	4.1/0.1–11.9	82.93
Hippocampal sclerosis	13	8	5	2.8/0.1–8.0	8.3/6.3–12.4	5.5/3.5–9.5	84.61
Tumor	42	27	15	4.0/0.1–13.0	7.6/5.0–14.0	3.6/0.1–13.0	85.71
GG	40	26	14	4/0.1–13.0	7.7/5.0–14.0	3.7/0.1–13.0	
DNT	1	1	0	1.2	7	5.8	
AG	1	0	1	1	3	2.0	
Glial scar	29	21	8	1.2/0.2–12.3	9.6/1.1–25.3	8.4/0.9–13.0	72.41
Encephalitis	27	15	12	1.6/0.2–12.0	8.8/1.2–24.5	7.2/1.0–12.5	70.37
No obvious pathological abnormality	23	12	11	4.5/0.2–13.3	11.1/1.3–26.8	6.6/1.1–13.5	69.56

Abbreviations: AG, angiocentric glioma; DNT, dysembryoplastic neuroepithelial tumor; F, female; GG, ganglioglioma; M, male.

A total of 41 cases belonged to FCD II, including 27 boys and 14 girls (Table 2). These cases accounted for 61.19% (41/67) of FCD patients. In the 41 FCD II cases, patient detailed clinical characteristics were seen in Table 2.

In FCD II patients, fourteen cases for FCD II a and 27 cases for FCD II b were included. More than half of the cases in FCD II group had epilepsy lesions in frontal lobe (24/41, 58.53% of FCD II cases). Six patients had lesions in the frontal lobe, accounting for 42.9% (6/14) of FCD II a cases. Eighteen patients had lesions in the frontal lobe, accounting for 66.7% (18/27) of FCD II b cases. Compared with FCD II a, FCD II b was more likely to locate in frontal lobe (*χ* ^2^ = 10.88, *p* < .01). Thirty‐four (Engel I:30, Engel II:4) patients were found with good prognosis, accounting for 82.93% of FCD II patients (Table 2). No statistical significance was found between FCD II and FCD I cases (*χ* ^2^ = 0.015, *p* > .05).

Postoperative seizure outcome of FCD was classified according to Engel. Engel I–II class was artificially regarded as an effective prognosis and Engel III‐IV as ineffective by us. The 67 patients with FCD were further divided into two subgroups according to duration for epilepsy history (<2 years or ≥2 years). We found the effective seizure outcome in cases with shorter duration of epilepsy (<2 years, 87.23%) was better than that in cases with longer duration (≥2 years, 60.00%), and the difference was statistically significant (*χ* ^2^ = 4.76, *p* < .05).

FCD II a had abnormal neurons (Figure 1c) but no balloon cells (BCs), and FCD II b had both abnormal neurons and BCs (Figure 1e). Balloon cells are located deeply in the cortex and subjacent white matter. This may be the reason for blurred gray‐white junction in the disease. Immunohistochemistry results showed abnormal neurons marked by NF (Figure 1d) and BCs were staining for Vimentin, Nestin (Figure 1f), and sometimes CD 68.

### 3.2 Hippocampal sclerosis

Thirteen cases had hippocampal sclerosis (boys: 8, girls: 5), and the mean age of onset was 2.8 years (range from 0.1 to 8.0). Patient detailed clinical characteristics were seen in Table 2. Eleven cases with hippocampal sclerosis had good seizure outcome (9 cases, Engel I; 2 cases, Engel II). The Engel class I seizure outcome of hippocampal sclerosis in the present study was 69.23% (9/13). The effective prognostic rate was 84.61% (11/13) (Table 2).

### 3.3 Tumor

Brain tumors were the second most prevalent histopathologic diagnosis, occurring in 18.11% (42 cases). In the present study, we found 3 types of tumors associated with epilepsy, including mixed neuronal‐glial tumor ganglioglioma (GG), dysembryoplastic neuroepithelial tumor (DNT), and angiocentric glioma (AG).

Ganglioglioma was the most frequent tumor type (*n* = 40, 95.23% of tumors) and represented lower grades (WHO grade I‐ grade II). Besides, one DNT and one AG were identified (Table 2). Thirty‐six cases in tumors had good seizure outcomes (Engel I:32, Engel II:4), and six cases had poor seizure outcomes. The Engel class I seizure outcome of tumor in the present study was 76.19% (32/42). The total effective prognostic rate in tumors was 85.71% (36/42) (Table 2).

Patient detailed clinical characteristics were seen in Table 2. Most lesions were located at temporal lobe (*n* = 25, 25/42, 59.52%). Of the other nontemporal lobe cases, twelve cases were located in the frontal lobe, two cases in the parietal lobe, two cases in the occipital lobe, and one cases in the temporal–occipital lobe.

**Figure 2:**
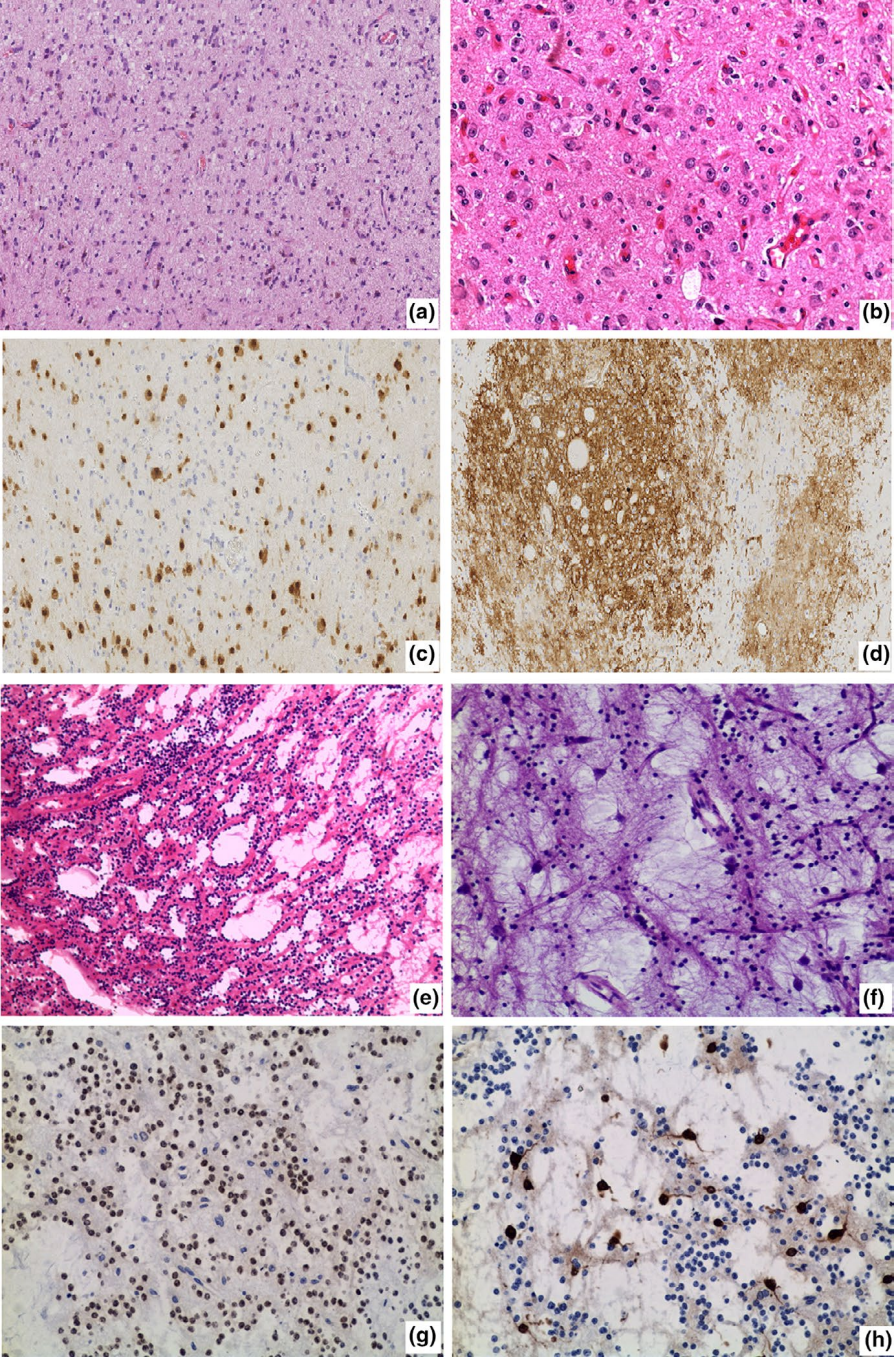
Histopathology of tumors (GG and DNT). (a and b) (GG) showed the hyperplasia of gliocyte. Some cells were in mild dysplasia. Numbers of neurons with different size and shape were distributed among hyperplastic gliocytes (HE × 100, ×200). (c) NeuN staining of the neurons (×100); (d) abundant CD34‐positive tumor aggregates can be identified within adjacent neocortex. (×100); (e and f) (DNT) showed that oligodendrocyte‐like cells were distributed like trees; some myxoid tiny cysts were formed, and neurons were floated in the cyst (E: HE × 100; F: HE × 200); (g) Olig‐2‐positive oligodendrocyte‐like cells (×200); (h) NeuN‐positive neurons floated in the cyst (×200)

Histopathologic examination of GGs revealed combination of neuronal and glial cells elements (Figure 2a,b). The neuronal areas were immunoreactive for NeuN (Figure 2c). Abundant CD34‐positive tumor aggregates can be identified within adjacent neocortex (Figure 2d). Histopathology of DNT showed findings typical of a glioneuronal neoplasm with a distinctive arrangement of oligodendroglial‐like cells and myxoid microcysts containing floating neurons (Figure 2e,f). The oligodendroglial‐like cells were positive for Olig‐2 (Figure 2g), and neurons floated in the cyst were positive for NeuN (Figure 2h). The microscopy of AG exhibited tumor cells arranged around vessels shaped like clumped chrysanthemums, and tumor cells were strongly and diffusely positive for GFAP and Nestin. Punctiformly positive expression of EMA was found in tumor cells.

### 3.4 Glial scars

A total of 29 cases were identified with glial scars, accounting for 12.50% of all cases (Table 1). They were often located in the temporal lobe or in multiple lobe. Of the 29 cases, fifteen cases were located in more than 2 lobes (with not well‐localized interictal and ictal EEG abnormalities), six cases in the temporal lobe, four cases in the parietal lobe, three cases in the occipital lobe, and one case in the insular lobe.

**Figure 3:**
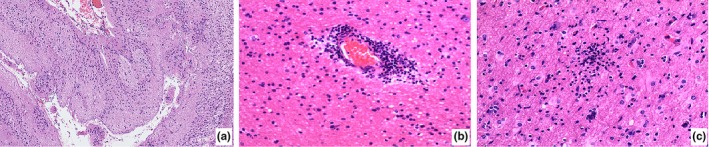
Histopathology of glial scars and encephalitis. (a) Focal cortex was missing, and glial scars were formed (HE × 100); (b) persistent perivascular T cell; (c) microglial nodule was formed (HE × 200)

Patient detailed clinical characteristics were seen in Table 2. The pathological features were shown in Figure 3a. Patient detailed clinical characteristics were seen in Table 2. Twenty‐four cases in glial scar cases had good seizure outcomes (Engel I: 20, Engel II:4), and five cases had poor seizure outcomes. The Engel class I seizure outcome of glial scar in the present study was 68.96% (20/29). The total effective prognostic rate in glial scar was 72.41% (Table 2).

### 3.5 Encephalitis

A total of 27 cases were identified with encephalitis, accounting for 11.63% of all cases (Table 1). Patient detailed clinical characteristics were seen in Table 2. The total effective prognostic rate in encephalitis was 70.37%. Twenty cases were identified as Rasmussen (RE, 8 boys and 12 girls, accounting for 74.07% of encephalitis). Histopathology of RE revealed shrinkage of the cortex and neuronal loss, capillary hyperplasia, and proliferation of glial cells. Persistent perivascular T cell (Figure 3b) and few microglial nodules were found (Figure 3c). The mean age of earliest seizure onset was 1.9 years old (range from 0.5 to 12.0). Twelve cases in RE had good seizure outcomes (Engel I:1, Engel II:11), and 8 cases had poor seizure outcomes. The total effective prognostic rate in RE was 60.00%.

### 3.6 Others types of pathology and seizure outcomes

A total of 23 cases with very small and only nonspecific findings, including distribution of amyloid bodies and gliosis, were observed. Among them, sixteen cases showed good seizure outcomes (recovery rate, 69.56%). Seven cases had Sturge–Weber syndrome, and the recovery rate was 57.14%. Seven cases had hamartomas, and the recovery rate was 85.71%; nine cases were diagnosed with tuberous sclerosis; four cases had gyrus deformation, three cases had gray matter heterotopia, and one case had a cyst with good seizure outcome.

## 4 DISCUSSION

The drug‐resistant seizures are accounting for ~30% of epilepsy. However, no effective treatment by medication has been developed and surgery was often needed for this special group of patients. Data have shown that the morbidity of drug‐resistant seizures was 15 times as low in adults, compared with in children, and moreover, 80% of these patients suffered from the disease before 18 years old. Up to 50% of pediatric surgery is due to epilepsy, which arises great concern for the study of pathological and mechanistic features of drug‐resistant seizures. In the study by Blumcke I (Blumcke et al., 2017), the most common categories were cortical malformations (mostly "isolated" FCD I or FCD II), found in 39.3% of the children patients, tumors in 27.2%, hippocampal sclerosis in 15%, glial scar in 5.8%, and encephalitis in 3.3%. In our study, 30.59% of patients had cortical malformations, tumors in 18.11%, glial scar in 12.50%, encephalitis (mainly RE) in 11.64%, no histopathologic diagnosis in 9.92%, and hippocampal sclerosis in 5.61%. In the present study, we report the histopathologic features of 232 Chinese children patients with drug‐resistant seizures receiving surgical resection. The 30.59% of patients had cortical malformations (mostly FCD, 94.36% of cortical malformations cases), which was lower than in Blumcke I et al study (Blumcke et al., 2017). But results showed that cortical malformations (mostly "isolated" FCD I or FCD I) was still the most important causes for children in the present study.

The effective prognostic rate for seizures in FCD I and FCD II group in this study showed no statistical difference as well as in literature Fauser et al. (2015). What was interesting about our result was the present retrospective study showed a trend of better prognostic rate of FCD (FCD I, 73.07%, II, 82.93%) than those reported in the literature, especially for pediatric patients with FCD I and II (FCD I, 72.72%, II, 76.47%) (Fauser et al., 2015). The duration for epilepsy history of our patients with FCD I and II was 4.80 years and 4.10 years, respectively, which were shorter than earlier report (FCD I, 13.8 years, 14.0 years). Although it was simply a trend of slightly better prognostic rate of FCD in the present study (with shorter seizure duration) than those reported in the literature, this prompted us to think about whether a shorter seizure duration was a predictor of favorable seizure outcomes in the present study. In view of this trend, we further studied the relationship between the duration of epilepsy and the seizure outcome of our FCD cases. We found the seizure outcome of FCD cases in our study with shorter duration of epilepsy (<2 years, 87.23%) was better than that in the FCD cases with longer duration (≥2 years, 60.00%). Similar to our results, a shorter seizure duration has been shown to be a predictor of favorable seizure outcomes in previous studies (Janszky et al., 2005; Ramantani et al., 2017). This implied operation should be did as early as possible on surgically remediable epilepsies, so the early surgery was probably a good choice.

Brain tumors were the second highest occurrence diagnosis in this study, occurring in 18.11% (42/232). The most frequent tumors were GG, accounting for 95.23% of tumors. Glioma patients were rare, probably because these patients rarely went to epilepsy centers for treatment. The expression of CD34 was not limited in the entity of tumors, but positive in cytoplasm or surrounding the nucleus, even positive around tumor edges (Deb et al., 2006; Ogiwara et al., 2010). In all of the 40 GG cases, 80% patients had CD 34 expressed like clouds or clustered, in accordance with previous reports (Giulioni et al., 2019; Piao et al., 2010). A previous study reported radiotherapy and chemotherapy are not necessary for GG with low grade after surgery (Luyken & Wiestler, 2002; Zentner et al., 1997). These patients did not receive any postoperative treatment in this group, and tumors did not recur.

In addition, the proportion of glial scars (5.8%) had been ranked the fourth in study of Blumcke et al (patients from 12 European countries) (Blumcke et al., 2017). But in this study, the glial scar was the third common histopathologic diagnosis occurring in 12.5% (29/232), most resulting from hypoxia/ischemia. As studies in china, they found that perinatal hypoxic–ischemic brain damage remains one of the most common complications in Chinese newborns (Piao et al., 2010). We thought it was the main cause for the high proportion of glial scar in our study. Glial scars in our group were most often located in multiple lobes (15/29, 51.72%). Engel class I outcome of glial scars was 68.96%. In contrast, only one case of tumor located in multiple lobes (41/42, 97.61%) and Engel class I outcome of tumor was 76.19%. Engel class I outcome of glial scars was worse than Engel class I outcome of tumor. Patients with glial scars are considered unsuitable for surgical operation because the range of lesions is usually very broad. The results also supported that surgical treatment of glial scars with multiple lobes was not recommended.

It was thought the total effective prognostic rate would be well for glial scars cases with a high incidence of relatively well‐localized interictal and ictal EEG abnormalities. Iida, Otsubo, Arita, Andermann, and Olivier (2005) reported 8 of patients with glial scars undergoing intraoperative electrocortical imaging (ECOG) guided underwent focal cortical resection, and 6 (75%) patients had a good prognosis for seizure control (Engel class I). Most of these patients had well‐localized and lesion‐concordant surface EEG findings in the presurgical work‐up. The Engel class I seizure outcome of glial scar in the present study was 68.96% (20/29), which was lower than it in Iida et al study (Iida et al., 2005). Of the 29 glial scar cases in this study, sixteen cases had wide extent of the lesion, not well‐localized interictal and ictal EEG abnormalities, suggesting the presence diffuse epileptogenic zones. Unfortunately, seizure outcome for them was poor.

## 5 CONCLUSION

In conclusion, MCD (most FCD) appeared to be the most common disease in Chinese children cases with drug‐resistant seizures. We speculated further that FCD patients with shorter duration of epilepsy seem to have a higher chance of good prognosis. The surgical treatment of glial scars with multiple lobes was not recommended. We hope that the data in the present study will enrich information on pathologic features in Chinese patients with drug‐resistant focal epilepsy.

## 6 LIMITATIONS

The limitations of the present study are that this was limited to a single center study. In this article, we focused on neuropathological descriptions and seizure outcome in pediatric cases. We did not get much information on neuropsychological findings and neuroimaging modalities in our cohort. We will consider more clinical information about these in our further study.

## CONFLICT OF INTEREST

The authors declare that they have no competing interests.

## AUTHOR CONTRIBUTIONS

Yao Kun participated in cytological diagnosis and drafted the manuscript. Duan Zejun and Yao Kun participated in the design of the study and cytological diagnosis. Zhou Jian, Zhai Feng and Liu Changqing helped in the selection and collection of tissues. Qi Xueling participated in the design of the study and performed the statistical analysis. All authors read and approved the final manuscript.

## Data Availability

Data availability requests can be directed to the corresponding author and made available as required in appropriate situations.
